# Role of the Insulin-like Growth Factor (IGF) Axis in Diseases

**DOI:** 10.3390/ijms242316969

**Published:** 2023-11-30

**Authors:** Claire M. Perks

**Affiliations:** Cancer Endocrinology Group, Bristol Medical School, Translational Health Sciences, Learning & Research Building, Southmead Hospital, Bristol BS105NB, UK; claire.m.perks@bristol.ac.uk

The insulin-like growth factor axis is a multifaceted, complex system that comprises two ligands, IGF-I and IGF-II, receptors (IGF-1R, IGF-IIR, insulin receptor isoforms IR-A and B, and hybrid receptors) six high affinity IGF-binding proteins (IGFBPs 1–6), and IGFBP proteases. The IGFs play an important role in normal growth and development through their modulation of cell proliferation, differentiation, glucose and lipid metabolism and cell survival. The main source of circulating IGF-I is the liver in response to growth hormone. Circulatory IGF levels are maintained through interaction with IGFBP-3 or IGFBP-5 and an acid labile subunit, a complex that increases the half-life of IGFs and maintains their concentration in the serum [1,2]. IGFs, however, are also produced locally in nearly every tissue of the body, for example, muscle and bone [3,4], exerting their effects through many signaling pathways, the two most critical ones being the RAS/MAPK and PI3K/AKT cascades [5]. Their biological activity is regulated by IGFBPs that are expressed in a tissue-specific manner. This regulation of IGFs by IGFBPs can have stimulatory or inhibitory effects on IGF action [6]. To add to the complexity of the system, the IGFBPs themselves can also exert actions that can either be dependent or independent of the IGF-1R. For example, IGFBP-1 and IGFBP-2 have been shown to intrinsically affect migration of Chinese hamster ovary cells and breast cancer cells, respectively [7,8], and IGFBP-5 altered osteoblast mitogenesis in a manner that was independent of IGFs or their receptors [9]. The way in which the components of the IGF axis interact is hugely influenced by the cellular *milieu,* for example, in vascular smooth muscle cells [10], and this is key to understanding tissue-specific differences in the regulation and activity of the IGFs. For example, levels of IGFs can be affected by numerous factors including cytokines, sex steroids and nutritional status [11]. Therefore, it is not surprising that dysregulation of the IGF axis has a significant influence on the risk, development, and progression of many diseases [12], such as Alzheimer’s disease [13] and cancer [14]. The importance of the IGF axis in normal growth and development and its contribution to pathological conditions underscores the importance of research to investigate ways in which the axis can be manipulated [15]. For example, assessing the potential of an IGF-1R receptor inhibitor, teprotumumab, in patients with thyroid eye disease [16]. In cancer research, targeting the IGF-1R has been difficult, with more than 15 inhibitors being evaluated in clinical trials that did not achieve the necessary clinical endpoints for regulatory approval. Several factors were proposed to explain their failure, including the absence of specific biomarkers to guide patient selection [17,18,19]. The contribution of the IGF axis to growth and development is indisputable, and continued research aimed at understanding its activity and how it is deregulated in pathological conditions is vital to develop ways through which it can be effectively targeted. Thus, researchers have conducted several studies, examining the role and impact of the IGF axis, specifically in cancer, autoimmune diseases, and fibrosis.2. An Overview of the Published Articles.

The complexity of the axis is highlighted in a detailed review presented by Kasprzak et al. that described the role of IGF isoform expression, its regulation, and role in tumor biology (contribution 1). The authors suggest that a better understanding of this additional layer may provide new ways to target the IGF axis. In terms of current strategies, Cao et al. discussed a rationale for targeting the IGF system in cancer, with a focus on breast cancer, the strategies that have been employed, the outcomes, and the lessons learnt (contribution 2). In line with this article, the review by Oliveres et al. focuses on metastatic colorectal cancer, analyzing the discoveries from previous attempts to target receptor tyrosine kinases, including the IGF-1R, and discussing how this information could be used for effective drug development in the future (contribution 3). Ngo et al. provide an overview of the role of IGF/IGF-1R in hepatocellular carcinoma (HCC) (contribution 4). Oliveres et al. and Ngo et al. similarly discuss resistance to tyrosine kinase inhibitors; Ngo et al. review this with respect to to cancer stemness and describe current therapies and those that could be available in the future, particularly in relation to hepatitis B virus-associated HCC (contribution 4). Obesity and type 2 diabetes (T2D) have a negative effect on the risk and progression of many cancer types [20]. One aspect of these conditions is the potentially harmful impact of higher-than-normal levels of glucose, known as hyperglycaemia. Koobotse et al. report that physiological levels of glucose, in contrast to hyperglycaemic levels, supported BRCA1 in restraining the lipogenic actions of IGF-I to regulate cell growth and determine that this was dependent on the association of BRCA1 with phosphorylated acetyl CoA carboxylase (ACCA) (contribution 5). In relation to other conditions in which the IGF axis plays an important role, Krieger et al. present a comprehensive review on the role of IGF-1R-stimulating antibodies in Graves’ orbitopathy. Graves’ orbitopathy is an autoimmune disease, recognized as swelling around the eye, that is initiated by an immune response against thyroid-stimulating hormone (TSH) and is treated with anti-inflammatory and surgical approaches. The authors provide evidence pertaining to the use of directly stimulating IGF-1Rs with autoantibodies in this condition. They describe a model of crosstalk that exists between TSHR and IGF-1R and discuss how this may be helpful when developing future therapies [21]. Finally, Nguyen et al., who had previously reported that insulin-like growth factor binding protein-5 (IGFBP-5) mediated the effects of fibrosis that occurred in conditions such as pulmonary fibrosis, use a transgenic model to further investigate the functional role of IGFBP-5 in the development of fibrosis in vivo (contribution 6).

This Special Issue highlights the diverse ways in which dysregulation of IGF signaling contributes to the development and progression of several diseases. It also emphasizes the need to further understand the complexity of the system to enable effective targeting.

## References

[1] LeRoith D., Holly J.M.P., Forbes B.E. (2021). Insulin-like growth factors: Ligands, binding proteins, and receptors. Mol. Metab..

[2] Baxter R.C. (2023). Signaling Pathways of the Insulin-like Growth Factor Binding Proteins. Endocr. Rev..

[3] Giustina A., Mazziotti G., Canalis E. (2008). Growth hormone, insulin-like growth factors, and the skeleton. Endocr. Rev..

[4] Kok H.J., Barton E.R. (2021). Actions and interactions of IGF-I and MMPs during muscle regeneration. Semin. Cell Dev. Biol..

[5] Hakuno F., Takahashi S.I. (2018). IGF1 receptor signaling pathways. J. Mol. Endocrinol..

[6] Holly J., Perks C. (2006). The role of insulin-like growth factor binding proteins. Neuroendocrinology.

[7] Frommer K.W., Reichenmiller K., Schutt B.S., Hoeflich A., Ranke M.B., Dodt G., Elmlinger M.W. (2006). IGF-independent effects of IGFBP-2 on the human breast cancer cell line Hs578T. J. Mol. Endocrinol..

[8] Jones J.I., Gockerman A., Busby W.H., Wright G., Clemmons D.R. (1993). Insulin-like growth factor binding protein 1 stimulates cell migration and binds to the alpha 5 beta 1 integrin by means of its Arg-Gly-Asp sequence. Proc. Natl. Acad. Sci. USA.

[9] Andress D.L., Birnbaum R.S. (1992). Human osteoblast-derived insulin-like growth factor (IGF) binding protein-5 stimulates osteoblast mitogenesis and potentiates IGF action. J. Biol. Chem..

[10] Duan C. (2002). Specifying the cellular responses to IGF signals: Roles of IGF-binding proteins. J. Endocrinol..

[11] Mancarella C., Morrione A., Scotlandi K. (2021). Novel Regulators of the IGF System in Cancer. Biomolecules.

[12] Song F., Zhou X.-X., Hu Y., Li G., Wang Y. (2021). The Roles of Insulin-Like Growth Factor Binding Protein Family in Development and Diseases. Adv. Ther..

[13] Galle S.A., Geraedts I., Deijen J., Milders M., Drent M. (2020). The Interrelationship between Insulin-Like Growth Factor 1, Apolipoprotein E epsilon4, Lifestyle Factors, and the Aging Body and Brain. J. Prev. Alzheimers Dis..

[14] Mancarella C., Morrione A., Scotlandi K. (2021). Unraveling the IGF System Interactome in Sarcomas Exploits Novel Therapeutic Options. Cells.

[15] Osher E., Macaulay V.M. (2019). Therapeutic Targeting of the IGF Axis. Cells.

[16] Subramanian P.S., Cho R.I., Kahana A. (2023). Efficacy of teprotumumab therapy in patients with long-duration thyroid eye disease. Curr. Opin. Ophthalmol..

[17] Crudden C., Girnita A., Girnita L. (2015). Targeting the IGF-1R: The Tale of the Tortoise and the Hare. Front. Endocrinol..

[18] Jentzsch V., Osipenko L., Scannell J.W., Hickman J.A. (2023). Costs and Causes of Oncology Drug Attrition With the Example of Insulin-Like Growth Factor-1 Receptor Inhibitors. JAMA Netw. Open.

[19] Lee J.S., Tocheny C.E., Shaw L.M. (2022). The Insulin-like Growth Factor Signaling Pathway in Breast Cancer: An Elusive Therapeutic Target. Life.

[20] Scully T., Ettela A., LeRoith D., Gallagher E.J. (2020). Obesity, Type 2 Diabetes, and Cancer Risk. Front. Oncol..

[21] Krieger C.C., Neumann S., Gershengorn M.C. (2020). TSH/IGF1 receptor crosstalk: Mechanism and clinical implications. Pharmacol. Ther..

